# Phosphoinositide-dependent kinase 1 (PDK1) in cancer: molecular insights and therapeutic strategies

**DOI:** 10.3389/fchem.2026.1767926

**Published:** 2026-03-03

**Authors:** Shatha Algheribe, Ayat Zagzoog, Mohamed Boudjelal, Imadul Islam

**Affiliations:** 1 Medical Research Core Facility and Platforms Department, King Abdullah International Medical Research Center (KAIMRC), King Saud bin Abdulaziz University for Health Sciences (KSAU-HS), Ministry of the National Guard – Health Affairs (MNGHA), Riyadh, Saudi Arabia; 2 Department of Pharmaceutical Analysis, King Abdullah International Medical Research Center (KAIMRC), King Saud bin Abdulaziz University for Health Sciences (KSAU-HS), Ministry of the National Guard – Health Affairs (MNGHA), Riyadh, Saudi Arabia

**Keywords:** allosteric modulation, cancer therapeutics, combination therapy, drug design, kinase signaling, PDK1 inhibitors, small molecules

## Abstract

3-Phosphoinositide-dependent protein kinase 1 (PDK1) has emerged as one of the most strategically positioned and paradoxically underexploited regulators within Pl3K. It is the main controller of the AGC kinase family, which includes AKT, S6K, SGK, and PKC isoforms. PDK1 is a central signaling hub downstream of the PI3K signaling pathway. It controls key cellular processes such as proliferation, metabolism, and survival by orchestrating activation-loop phosphorylation. Aberrant activation of PDK1 facilitates tumor initiation, progression, and therapeutic resistance in various cancer types. Scientists have not been able to develop small-molecule inhibitors that are as selective and work as well in the clinic as they do for other kinases. This is mostly because the ATP-binding site is highly conserved, while PDK1’s structure is very dynamic. This review summarizes recent progress in comprehending PDK1’s structure, regulation, and its function in oncogenic (cancer-promoting) signaling. We discuss medicinal chemistry strategies like ATP-competitive, allosteric, and dual-site inhibition, as well as rational polypharmacology and combination approaches to overcome pathway redundancy. We also discuss how far we have come in identifying biomarkers to help us select patients and monitor their responses. These efforts make PDK1 a promising but underused target for therapy. New opportunities are emerging to use it for diseases beyond cancer, such as inflammatory, metabolic, and neurological diseases.

## 1 Introduction

The 3-phosphoinositide-dependent protein kinase-1 (PDK1) is an important enzyme in the PI3K signaling pathway, which is a major regulatory network that controls cell growth, metabolism, proliferation, and survival (Han et al., 2023). PDK1 activates a subset of protein kinases known as AGC kinases (including AKT and S6K), which are vital in regulating numerous essential cellular activities (Gagliardi et al., 2018). In this capacity, PDK1 transmits extracellular growth and nutrition cues into intracellular mechanisms that regulate cell division, metabolic adaptability, and survival responses (Han et al., 2023; Wang et al., 2017).

PDK1 is especially significant in oncology, where dysregulated activation of the PI3K pathway commonly arises due to activating PIK3CA mutations, hyperactivation of receptor tyrosine kinases, or the loss of the tumor suppressor PTEN, which typically counteracts PI3K signaling by dephosphorylating phosphatidylinositol (3,4,5)-trisphosphate (PIP_3_) (Bamodu et al., 2020). In addition to its oncogenic relevance, PDK1 is essential for insulin signaling, glucose regulation, and immune cell activation, underscoring its significance in both pathological and physiological contexts (Maurer et al., 2009; Raimondi and Falasca, 2011).

**FIGURE 1:**
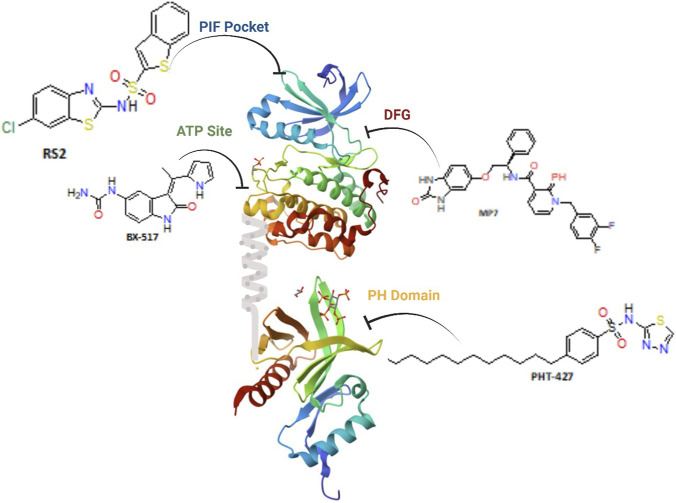
Illustrative PDK1 structure emphasizing druggable cavities. Ribbon depiction of full-length PDK1 exhibiting small-molecule ligands associated with the ATP site (BX-series inhibitor), the allosteric PIF pocket (sulfonamide RS2), a DFG-out–stabilizing pocket (pyridonyl derivative MP7), and the PH domain (PHT-427), demonstrating the primary binding modalities utilized for PDK1 inhibition.

PDK1 consists of a bilobal kinase domain that enables catalytic phosphorylation, a C-terminal pleckstrin homology (PH) domain that interacts with phosphoinositide lipids at the plasma membrane, and a conserved PDK1-interacting fragment (PIF) pocket that facilitates the docking and phosphorylation of downstream AGC kinase substrates (Levina et al., 2022). Figure 1 illustrates that these domains operate synergistically to regulate substrate recognition, localization, and activation (Sacerdoti et al., 2023). Although recent structural and mechanistic investigations have improved the understanding of PDK1 regulation and function, facilitating inhibitor design (Gagliardi et al., 2018), medication development remains challenging (Xu et al., 2024). The notable conservation of the ATP-binding site across the kinome, along with compensatory signaling through parallel growth pathways, has hindered the development of selective and durable PDK1 inhibitors (Jiang et al., 2021; Sadeghian et al., 2025). Consequently, innovative therapy approaches are currently focusing on bivalent and dual-site inhibitors, as well as targeted protein degradation techniques, which may overcome these limitations and enhance the efficacy of PDK1-targeted therapies (Ma et al., 2025).

### 1.1 Kinase signaling and cancer progression

Cancer continues to be a predominant global health concern, resulting in over 10 million fatalities in 2020, accounting for approximately one-sixth of all deaths globally (Sestilo and Rapposelli, 2019; World Health Organization, 2025). Despite advancements in precision medicine and immunotherapies, cancer persists in demonstrating considerable variability and adaptability (Fruman et al., 2017; Gillet et al., 2013). Variation in mutations, cell origins, and environments gives rise to many cancer subtypes. Characteristics encompass unregulated proliferation, evasion of apoptosis, invasion, metastasis, and resistance to pharmacological agents (Liu et al., 2015; Tandel et al., 2019), frequently associated with the dysregulation of protein kinases (Fruman et al., 2017).

In the past 20 years, protein kinases have become key drug targets for discovery and therapeutic innovation. Small-molecule kinase inhibitors are among the fastest-growing therapies, reflecting their critical function in cellular regulation. By 2025, over 85 kinase inhibitors are expected to gain the U.S. Food and Drug Administration approval (Roskoski R., 2025), with 42 launched from 2018 to 2023, emphasizing their clinical value (Bhullar et al., 2018; Busschots et al., 2012; Latham et al., 2024). ATP-competitive inhibitors targeting Breakpoint Cluster Region-Abelson (BCR-ABL), Epidermal Growth Factor Receptor (EGFR), and the B version of the Raf kinase family discovered in a rapidly accelerated fibrosarcoma (BRAF) have had a significant clinical impact (Anaya et al., 2025). Yet resistance, toxicities, and varied efficacy highlight the need for new kinase targets (Jiao et al., 2018).

### 1.2 PDK1 within the PI3K/AKT pathway

The PDPK1 gene encodes 3-phosphoinositide-dependent protein kinase-1 (PDK1), a key serine/threonine kinase in the PI3K/AKT signaling pathway (Chan and Tsichlis, 2001; Jiang et al., 2021). PDK1 acts as a “master kinase,” adding phosphate groups to the activation-loop (T-loop) residues of many AGC family members (Bayascas, 2008; Jiang et al., 2021). When class I PI3Ks are activated, PIP_2_ is converted to PIP_3_ at the membrane (Falasca and Fyffe, 2013; Maurer et al., 2009). This brings PDK1 and AKT together via their PH domains, enabling PDK1 to phosphorylate AKT at Thr-308 to activate it (with Ser-473 phosphorylated by the mTORC2 complex, which supports cell growth signaling).

**FIGURE 2:**
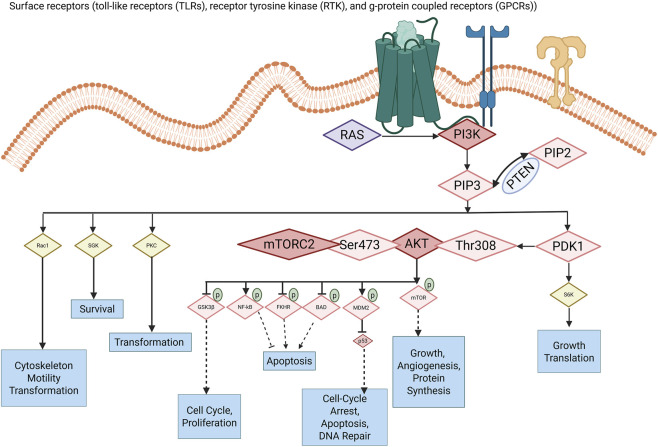
PDK1 signaling in the PI3K/AKT pathway, highlighting oncogenic roles. Surface receptors (TLRs, RTKs, and GPCRs) activate RAS and class I PI3K, resulting in the conversion of PIP_2_ to PIP_3_, which is counteracted by PTEN. PIP_3_ recruits PDK1 and AKT to the plasma membrane, where PDK1 phosphorylates AKT at Thr308 and mTORC2 phosphorylates AKT at Ser473, resulting in fully active AKT that facilitates cell survival, proliferation, growth, angiogenesis, and DNA damage responses through substrates including GSK3β, NF-κB, FOXO, BAD, MDM2, and mTOR. AKT-dependent PDK1 signaling is demonstrated via the conventional PDK1→AKT→downstream effector pathway, which includes cell-cycle progression, anti-apoptosis, and transformation. PDK1 signaling, independent of AKT, is illustrated through the direct phosphorylation of several AGC kinases, including S6K, SGK, PKC, and Rac1-regulated pathways, which facilitate protein translation, cytoskeletal remodeling, motility, transformation, and survival without reliance on AKT.

Besides AKT, PDK1 also activates other members of the AGC kinase family, a group of related enzymes involved in cell growth, survival, metabolism, and motility. The activity of PDK1 is tightly controlled (Yang et al., 2022). For example, when S6K1 (an AGC kinase, downstream of mTORC1) adds a phosphate to the PH domain of PDK1, it promotes binding to 14-3-3 proteins (a regulatory protein), leading to PDK1 pairing with itself (dimerization) and moving away from cell membrane phospholipid (PIP_3_), which reduces AKT activation (Jiang et al., 2022). This type of feedback regulates AKT activities during stimulation by another enzyme complex, mTORC1 (Chan and Tsichlis, 2001; Jiang et al., 2021). Figure 2 shows regulators in the PI3K/Akt pathway, with arrows indicating activation (turning on) or inhibition (turning off). Akt signaling promotes key processes in cancer, such as tumor cell growth, survival, proliferation, changes in immune response, metabolism, and angiogenesis (He et al., 2021).

In cancer, dysregulation of PDK1 signaling is recognized as a cause of oncogenesis (Feldman et al., 2005; Zheng et al., 2023). Research on breast cancer indicates that the gene for PDPK1 is often found in a higher amplified or overexpressed form, frequently alongside upstream oncogenic modifications within the PI3K pathway (Wang et al., 2024). These encompass activating mutations in PIK3CA, the loss of the tumor suppressor PTEN, and the amplification or overexpression of ERBB2/HER2, all of which culminate in increased PDK1 signaling output (Medina, 2013; Park et al., 2023; Piri et al., 2024).

Increased PDK1 expression augments AKT activation, stimulates cellular proliferation, heightens tumor aggressiveness, and fosters resistance to PI3K-targeted therapies (Yang et al., 2017; Yang et al., 2019). Persistent hyperactivation of PDK1, particularly by prolonged phosphorylation at Ser241, is commonly detected in various cancer types and correlates with unfavorable clinical outcomes and aggressive disease characteristics (Bamodu et al., 2020). The genetic deletion of PDK1 in PTEN-deficient tumor models significantly inhibits tumor growth, highlighting its pivotal function as a therapeutic target. Aberrant PI3K–AKT–mTOR signaling, instigated by PTEN loss, activating PI3K mutations, or hyperactive receptor tyrosine kinases (RTKs), constitutes a pivotal oncogenic occurrence, with PDK1 serving as a crucial mediator for relaying oncogenic signals to downstream AGC kinases (Yang et al., 2017; Yang et al., 2019).

In accordance with this model, genetic investigations reveal that a partial deficiency of PDK1 substantially inhibits tumorigenesis in PTEN-deficient environments (Chen et al., 2017; Haddadi et al., 2018), whereas diminished PDK1 expression in PTEN^+/−^ mice significantly postpones spontaneous tumor formation (Bayascas et al., 2005). In Braf^V600E^; Cdkn2a^−/−^ melanoma models, the deletion of PDK1 significantly hinders tumor growth and metastatic advancement, despite the presence of functional PTEN signaling (Scortegagna et al., 2015). In cerebral tissues, the suppression of PDK1 reduces the hypertrophic reaction induced by PTEN deficiency, thereby underscoring the context-specific carcinogenic functions of PDK1 (Chalhoub et al., 2009).

Clinically, heightened PDK1 expression and sustained phosphorylation at Ser241 correlate with unfavorable prognosis in patients with breast, prostate, and glioblastoma cancers, highlighting the importance of PDK1 activation status as both a biomarker for disease severity and a potential predictor of treatment efficacy (Hossen et al., 2015).

### 1.3 Rationale for targeting PDK1

PDK1 is crucial in carcinogenesis by enabling tumor initiation and progression through enhancing cell motility, invasion, and adaptation to unfavorable microenvironmental conditions, such as hypoxia and nutrient scarcity (Emmanouilidi and Falasca, 2017; Sestito et al., 2018). Comprehensive experimental evidence indicates that PDK1 governs various stages of the metastatic cascade, encompassing invadopodia development, increased matrix metalloproteinase activity, and dynamic reorganization of the actin cytoskeleton (Baldi et al., 2025; Xie et al., 2006). These activities are facilitated by both kinase-dependent and kinase-independent methods, involving downstream effectors including ROCK1, MRCKα, and PLCγ1 (Garcia-Viloca et al., 2022). In ovarian cancer, PDK1 overexpression is significantly correlated with metastatic spread, chemoresistance, and reduced patient survival (Siu et al., 2020). PDK1 mechanistically promotes cancer cell adhesion, invasion, and angiogenesis by activating the α_5_β_1_ integrin axis and the downstream JNK/IL-8 signaling pathways (Siu et al., 2020). The findings jointly designate PDK1 as a crucial regulator of tumorigenesis, metastatic potential, and therapeutic resistance, underscoring its importance as a primary target for anti-cancer interventions (Wang et al., 2024).

The justification for focusing on PDK1 is strengthened by the inherent heterogeneity and adaptive potential of cancer cells. Notwithstanding considerable progress in precision oncology, targeted treatments, and immunotherapies, therapeutic resistance persists, fueled by genetic heterogeneity and the constant reconfiguration of intracellular signaling networks (Fruman et al., 2017; Gillet et al., 2013). Deviant kinase signaling perpetuates fundamental characteristics of cancer, including unregulated growth, resilience to metabolic and genotoxic stress, invasion, and metastatic spread (Firnau and Brieger, 2022; Manning and Dibble, 2024). PDK1, as a principal regulator of AGC kinases, serves a pivotal role in these signaling networks, with its dysregulation directly linked to tumor growth and resistance to therapy (Zheng et al., 2023). Thus, PDK1 serves as a compelling and mechanistically validated target for the development of anticancer therapeutics (Emmanouilidi and Falasca, 2017).

Early attempts to block PDK1 with a drug faced significant challenges. Early compounds such as BX-320, BX-795, and BX-912, lacked selectivity in cellular contexts (Dangelmaier et al., 2014; Knight, 2011). A deeper understanding of PDK1’s structure and the new drug design method led to the development of more selective inhibitors, such as GSK2334470, which demonstrates sub-10 nM potency and increased selectivity (Najafov et al., 2011). New approaches that block regions such as the PIF pocket have also been developed. These offer ways to stop PDK1 activity beyond simply blocking its ATP site (Schulze et al., 2016; Wilhelm et al., 2012). These new inhibitor types, along with combination therapies and biomarker use, are revitalizing interest in PDK1 inhibitors for cancer treatment (Chandrasekhar et al., 2018; Schulze et al., 2016; Wilhelm et al., 2012).

Recent evidence underscores the potential benefits and obstacles linked to the targeting of PDK1 (Park et al., 2023). The structural similarities of kinase ATP-binding sites, the context-dependent effects of PDK1 inhibition, and compensatory signaling systems undermine the effectiveness of single-agent inhibitors. The area is progressively transitioning towards combination therapy strategies, the creation of multitarget ligands, and biomarker-driven patient selection methods to address adaptive resistance and enhance clinical outcomes (Sadeghian et al., 2025). Beyond oncology, PDK1 inhibition is being explored for potential applications in allergic conditions and antifungal drug development, indicating its broader therapeutic potential (Sestito and Rapposelli, 2019). Taken together, these recent advancements affirm PDK1’s pivotal role in tumor initiation, progression, and resistance to therapy, underlining its promise as a drug target. This review consolidates existing knowledge of PDK1’s biological functions and offers an updated perspective on its inhibition through innovative medicinal chemistry strategies, including the development of ATP-competitive, allosteric, and substrate-based inhibitors, as well as techniques targeting protein–protein interactions. Additionally, it emphasizes the integration of high-throughput screening, structure-based drug design, polypharmacology, combination therapies, and predictive biomarker strategies, guiding the clinical translation of PDK1-targeted agents and informing the rationale for designing next-generation inhibitors in precision oncology.

## 2 Structure and regulation of PDK1

PDK1 is a 556-amino-acid serine/threonine kinase whose structural organization underpins its function as a central signaling integrator within the AGC kinase family. The enzyme has a bilobal catalytic domain, a PH domain at the C-terminus, and a conserved PIF pocket that facilitates substrate docking and enables allosteric regulation (Barile et al., 2012; Xu et al., 2019). Key residues, such as Lys111, Thr222, Ala162, and the gatekeeper Met120, define the catalytic site. These residues help ATP bind, enhance selectivity, and stabilize the active conformation (Islam et al., 2007a; Leroux and Biondi, 2023; Schulze et al., 2016). Dynamic interactions within the molecule, membrane recruitment via PIP_3_ binding, and regulatory phosphorylation events, such as the one at Ser393, which causes conformational activation, all control PDK1 activity (Wu et al., 2009; Xu et al., 2024). PDK1 acquires significant flexibility via interdomain interactions, allowing it to precisely regulate downstream kinases, including AKT, S6K, and PKC isoforms, in both spatial and temporal contexts (Levina et al., 2022). This complexity in architecture and regulation not only explains why it is so important for cell signaling, but it also gives us many ways to target it for treatment, such as ATP-competitive, allosteric, and dual-site (Garcia-Viloca et al., 2022; Sadowsky et al., 2011).

### 2.1 Domain architecture and critical residues

PDK1’s multi-domain structure is central to AGC kinase signaling (Sadeghian et al., 2025; Xu et al., 2019). PDK1 contains a bilobal catalytic domain and a C-terminal PH domain. This pH domain binds membranes via phosphoinositides, particularly phosphatidylinositol 3, 4, 5-trisphosphate (PIP_3_) (Barile et al., 2012). A conserved PIF (PDK1-Interacting Fragment) pocket connects these domains, docks site substrates, and recognizes AGC kinases (Schulze et al., 2016). This architecture integrates kinase structure with signaling. The glycine-rich loop and Asp205 enhance flexibility, facilitating domain transitions and the dynamic conformation of PDK1 (Xu et al., 2024). These characteristics combined affect activation and regulation. Activation Mechanisms and Regulation of Conformational.

The activity of PDK1 is regulated by a comprehensive framework that includes interactions with the autoinhibitory domain, phosphoinositide binding, and phosphorylation of the activation loop, which collectively influence kinase conformation, localization, and signaling output (Meng et al., 2025). The involvement of PIP_3_-rich membranes alleviates autoinhibition, facilitates PDK1 dimerization and trans-autophosphorylation at Ser241, and dynamically modifies substrate and adaptor contacts to enhance AGC kinase signaling (Shaw and Burke, 2025). Prolonged lipid-dependent activation and continuous Ser241 phosphorylation, rather than mere PDPK1 expression, characterize pathological PDK1 addiction in cancer and establish a molecular foundation for its prognostic and therapeutic significance (Levina et al., 2022; Sacerdoti et al., 2023; Shaw and Burke, 2025; Xu et al., 2024).

### 2.2 Autoinhibition and conformational regulation of PDK1

PDK1 activity is rigorously regulated by an inherent autoinhibitory mechanism, wherein interactions among the pleckstrin homology (PH) domain, the kinase domain, and the intervening regulatory linker maintain an inactive cytosolic conformation and restrict substrate accessibility. Structural and computational analyses demonstrate that this closed conformation inhibits productive PDK1 dimerization and restrains activation-loop phosphorylation until suitable upstream signals are activated, thereby linking structural rearrangement to functional activation instead of allowing constitutive signaling (Mulpuri et al., 2025; Park et al., 2023). The phosphorylation of C-terminal regulatory residues, including Ser393, has been shown to reduce autoinhibitory contacts and promote conformational release; nevertheless, these modifications act as modulators rather than primary activators (Xu et al., 2024).

### 2.3 Lipid binding as a spatial and conformational modulator

The binding of phosphoinositides formed by PI3K—mainly phosphatidylinositol-(3,4,5)-trisphosphate (PIP_3_) and PI(3,4)P_2_ to the PH domain facilitates the recruitment of PDK1 to the plasma membrane, inducing a change from an autoinhibited monomer to an active, face-to-face dimeric form (Levina et al., 2022; Xu et al., 2024). The lipid-induced dimerization significantly enhances trans-autophosphorylation and reveals the PIF pocket, promoting effective binding to AGC kinases devoid of PH domains, including S6K, SGK, and RSK (Sacerdoti et al., 2023; Wu et al., 2009). Phosphoinositide binding serves mostly as a signal-amplifying spatial organizer, improving substrate colocalization and route flow instead of directly augmenting intrinsic catalytic turnover (Heras-Martínez et al., 2019).

#### 2.3.1 Impact of lipid-mediated activation on PDK1 protein interactions

Lipid-dependent membrane recruitment also alters the PDK1 interactome by indirectly modulating connections with scaffold and inhibitory partners. Although PDK1–substrate interactions (AKT, PKC, SGK, RSK) are significantly augmented by membrane colocalization and PIF-pocket exposure, the impact of phosphoinositides on interactions with regulatory proteins like 14-3-3 and Freud-1 (CC2D1A) seems to be subordinate to alterations in localization and conformation, rather than a direct lipid-binding effect (Park et al., 2023; Xu et al., 2024). Current data thus endorse a paradigm wherein lipid interaction alters the equilibrium in favor of signaling-competent PDK1 complexes, rather than functioning as a binary molecular switch for specific adaptor proteins (Torres et al., 2020).

#### 2.3.2 Phosphorylation of the activation loop and pathological dependence on PDK1

The phosphorylation of Ser241 in the activation loop is essential for the catalytic activity of PDK1 and occurs via lipid-induced trans-autophosphorylation during dimerization, rather than through an upstream kinase (Levina et al., 2022; Xu et al., 2024). This alteration stabilizes the active conformation of the kinase domain, aligns catalytic residues, and facilitates prolonged phosphorylation of downstream AGC kinases following brief membrane interaction (Sadowsky et al., 2011). While Ser241 phosphorylation is consistently observed in numerous normal cells, its sustained presence during chronic PI3K pathway activation is associated with aggressive tumor characteristics and unfavorable prognosis, indicating a reliance on functional PDK1 rather than mere overexpression, thereby establishing p-Ser241 as a dynamic biomarker of pathway dependency (Domrachev et al., 2021; Zhang et al., 2017).

### 2.4 Structural insights relevant to drug design

The structure and conformational changes of PDK1 identify significant targets for drug design, including the residues Lys111, Ala162, Ser160, Met120, and the PIF-pocket, all essential for inhibitor interaction (Sadeghian et al., 2025). These characteristics facilitate the systematic design of ATP-competitive, allosteric, and dual-site inhibitors (Garcia-Viloca et al., 2022; Nagashima et al., 2011; Sadowsky et al., 2011).

The adaptability of the glycine-rich loop and PH domain allows for the targeting of various PDK1 conformations, enhancing inhibitor selectivity and aiding in efficient drug design (Kumar et al., 2025). This conformational plasticity facilitates the creation of compounds that more accurately inhibit PDK1 activity, reducing compensatory signaling and improving target selectivity (Yao et al., 2020). These structural features therefore enhance rational drug design strategies for PDK1 in oncology and other diseases, establishing a direct link to therapeutic applications (Xu et al., 2024).

## 3 Biological role of PDK1 in cancer

**FIGURE 3:**
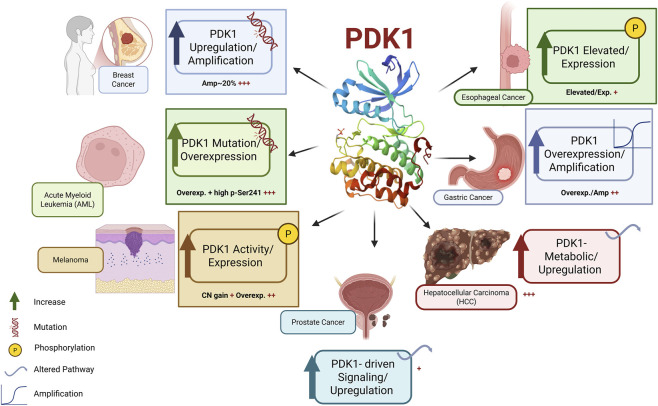
Variations of phosphoinositide-dependent kinase 1 (PDK1) in human malignancies. Schematic representation depicting the recurrent processes of PDK1 deregulation in several tumor types. PDK1 functions as a pivotal signaling hub downstream of PI3K, assimilating lipid-derived signals through its pleckstrin homology (PH) domain and activating AGC family kinases via its kinase domain and PIF pocket. In various malignancies, such as breast cancer, acute myeloid leukemia, melanoma, prostate cancer, gastric cancer, e sophageal cancer, and hepatocellular carcinoma, PDK1 is inappropriately activated via gene amplification, copy-number gain, overexpression, increased Ser241 phosphorylation, or pathway reconfiguration. These modifications induce oncogenic effects, including persistent PI3K–AKT–mTOR signaling, AKT-independent AGC kinase activation, metabolic reprogramming, invasion, and resistance to therapy. The pervasive and context-specific deregulation of PDK1 highlights its function as a systems-level regulator and a significant therapeutic target in several genetically and phenotypically distinct malignancies.

Phosphoinositide-dependent kinase 1 (PDK1) serves as a pivotal oncogenic signaling hub, with its dysregulation implicated in tumor initiation, development, and unfavorable clinical outcomes across several malignancies Figure 3. The hyperactivation of the PI3K–PDK1–AKT axis is a prevalent characteristic of cancer, resulting from PDPK1 gene amplification, overexpression, increased phosphorylation, or adaptive pathway reconfiguration (Bamodu et al., 2020). In breast cancer, increased PDK1 often coincides with PIK3CA mutations and stimulates PI3K pathway activity, facilitating malignant transformation and tumor sustenance (Ji et al., 2023; Maurer et al., 2009). Elevated PDK1 expression is common in acute myeloid leukemia, metastatic and castration-resistant prostate cancer, esophageal squamous cell carcinoma, gastric cancer, and hepatocellular carcinoma, where it is associated with advanced disease stage, metabolic reprogramming, and diminished patient survival (An et al., 2020; Bamodu et al., 2020; Lei et al., 2022; Luo et al., 2022; Nepstad et al., 2020; Starzyńska et al., 2020; Wang et al., 2017; Zabkiewicz et al., 2014). Figure 3 illustrates that these recurrent modifications establish PDK1 as a pivotal nexus for oncogenic signaling across several tissue contexts, facilitating tumor proliferation, invasion, and therapeutic resistance, thereby underscoring its significance as a widely actionable target in cancer biology (Xu et al., 2019).

### 3.1 Regulation of AGC kinases and oncogenic pathways

PDK1 is primarily known for activating AKT by phosphorylating Thr308 (Yang et al., 2022). This modification is essential for full AKT activity (Lu et al., 2010). Activated AKT promotes cell survival, glucose metabolism, and angiogenesis. Cancer frequently exploits these processes (Irazoqui et al., 2023). PDK1 also phosphorylates other AGC kinases, including S6K, SGK, and various PKC isoforms (Levina et al., 2022). This action broadens its influence to cell growth, cell cycle progression, migration, invasion, and metabolic adaptation (Tsai et al., 2022; Wang et al., 2021).

PDK1 is often elevated in cancers with PTEN loss or PI3K hyperactivation, leading to sustained AKT signaling and downstream pathway activation. Genetic models show their essential role in PTEN-loss–driven tumorigenesis. These findings highlight PDK1 as a key component of oncogenic signaling networks (Chen et al., 2017; Gagliardi et al., 2018).

### 3.2 PDK1 in tumorigenesis and therapeutic resistance

PDK1 directly contributes to tumor progression by facilitating epithelial–mesenchymal transition (EMT), stem cell–like traits, and therapy resistance (Chen et al., 2018; Chen et al., 2017). In breast cancer, elevated PDK1 activity is associated with resistance to trastuzumab and tamoxifen (Wang et al., 2022). In glioblastoma, inhibiting both PDK1 and Aurora-A reduces tumor cell survival. This phenomenon underscores its involvement in adaptive resistance mechanisms (Emmanouilidi and Falasca, 2017; Falasca and Fyffe, 2013). Furthermore, PDK1 facilitates carcinogenic characteristics via AKT-independent pathways (Vasudevan et al., 2009). In BCR–ABL–driven hematological malignancies, pharmacological or genetic suppression of PDK1 diminishes tumor cell survival with negligible alterations in AKT activity, suggesting dependence on alternate downstream effectors. These data indicate that PDK1 maintains cancer cell viability and proliferation via non-AKT AGC kinase signaling pathways. These data collectively affirm the role of PDK1 as a significant promoter of tumor growth, invasion, and adaptive survival in response to therapeutic pressure (Castel et al., 2016; Di Blasio et al., 2017; Emmanouilidi and Falasca, 2017; Manning and Toker, 2017; Raimondi et al., 2012).

### 3.3 Clinical correlations and prognostic relevance

Clinically, PDK1 activity is linked to poor outcomes in many cancer types. Elevated phosphorylation at Ser241 correlates with a worse prognosis in breast, prostate, glioblastoma, and pancreatic cancers. This highlights its potential as a prognostic biomarker (Huang et al., 2024). Its role in therapy resistance supports targeting PDK1 in clinical settings. Inhibiting PDK1 may enhance sensitivity to chemotherapy, targeted therapies, or combination regimens. In summary, PDK1’s key role in oncogenic signaling, tumor progression, and drug resistance makes it a significant therapeutic target and predictor of clinical outcomes (Falasca and Fyffe, 2013). These results indicate that PDK1 is a central signaling hub linking PI3K activation to downstream AGC kinases. These kinases are important for tumor growth, metabolism, and treatment resistance. Phosphorylation at Ser241 is a key marker of activation and may indicate how well a cancer will respond to treatment (Han et al., 2023).

## 4 Small molecule inhibition strategies

**FIGURE 4:**
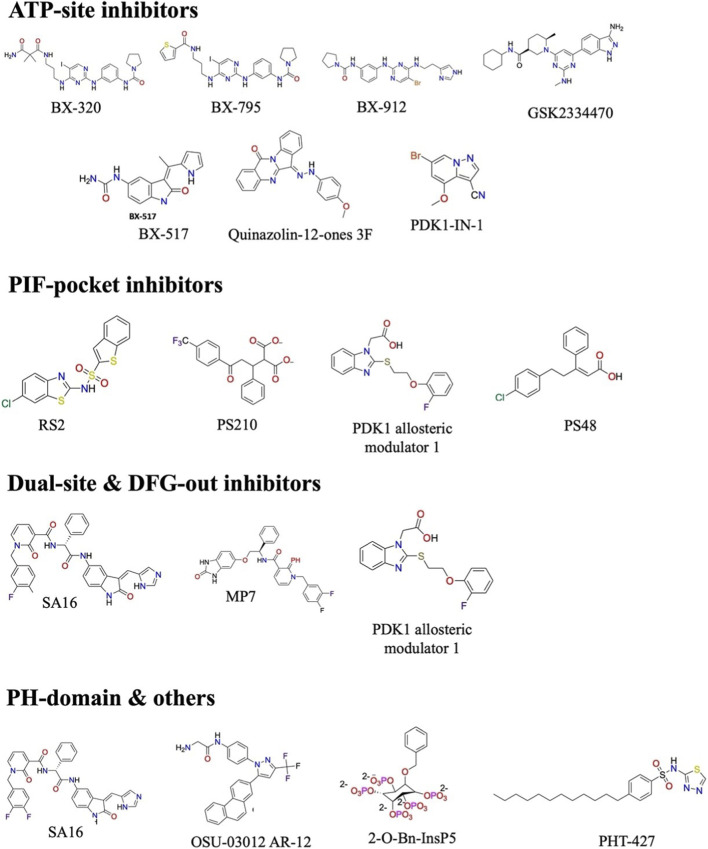
Exemplary small-molecule PDK1 inhibitors categorized by binding mechanism. The chemical structures of ATP-site inhibitors (BX-320, BX-795, BX-912, GSK2334470, BX-517, quinazolin-12-ones 3F, and PDK1-IN-1), PIF-pocket allosteric inhibitors (RS2, PS210, PS48, and related modulators), dual-site/DFG-out inhibitors (SA16, MP7, and PDK1 allosteric modulators), and PH-domain/other ligands (SA16, OSU-03012/AR-12, 2-O-Bn-InsP5, and PHT-427) exemplify the primary chemotypes employed to target specific regulatory pockets within PDK1.

Pharmacological investigations focused on PDK1 have produced a varied array of small compounds, encompassing ATP-competitive, allosteric, dual-site, and multi-target strategies (Fu et al., 2025). These methodologies unequivocally illustrate the structural intricacy and regulatory adaptability of PDK1 (Xu et al., 2024). This variability presents a substantial opportunity for therapeutic intervention. Figure 4 illustrates the most efficacious and mechanistically varied PDK1 inhibitors, classified by their primary binding sites on the kinase (Llorente et al., 2025). ATP-competitive inhibitors, including BX- and GSK-series medicines, engage with the catalytic ATP-binding cleft but often have limited selectivity and dose-dependent toxicity (Olivieri et al., 2022). PIF-pocket inhibitors, such as sulfonamide compounds, disrupt substrate binding without directly impairing catalytic activity, offering an alternative route with improved selectivity (Leroux and Biondi, 2023). Dual-site and DFG-out inhibitors, such as pyridonyl drugs, stabilize inactive kinase conformations by simultaneously binding to the ATP site and adjacent regulatory pockets, hence reducing compensatory activation of AGC kinases (Thakur et al., 2022). Supplementary modulators targeting the PH domain or non-canonical sites interfere with lipid-dependent membrane recruitment and conformational activation, highlighting innovative alternatives to conventional kinase inhibition (Barile et al., 2012; Busschots et al., 2012; Fu et al., 2025; Garcia-Viloca et al., 2022; Rettenmaier et al., 2014).

### 4.1 ATP-competitive inhibitors and limitations

ATP-competitive inhibitors bind the conserved ATP-binding pocket of PDK1 and directly compete with ATP at the catalytic site (Jug and Ilaš, 2025). Early compounds such as BX-320, BX-795, and BX-912 showed high potency, with IC_50_ values ranging from 11 to 30 nM (Feldman et al., 2005; Islam et al., 2007). However, these inhibitors lacked selectivity. They often affected other kinases, including TBK1 and IKKε. This reduced their therapeutic potential and highlighted the challenge of targeting a conserved active site (Knight, 2011; Peifer and Alessi, 2008).

The introduction of GSK2334470 marked a significant advancement. This ATP-competitive inhibitor shows exceptional selectivity (Yang et al., 2017). With an IC_50_ of about 10 nM, GSK2334470 is roughly 1000-fold more selective across a panel of 93 kinases (Najafov et al., 2011). It effectively inhibits phosphorylation of downstream substrates such as SGK and S6K. Its effects on AKT are context-dependent and reflect the complex substrate-specific interactions of PDK1 (Knight, 2011; Medina, 2013). Despite these improvements, challenges remain. Compensatory signaling pathways and potential toxicity from residual off-target effects continue to pose problems. The situation emphasizes the need for alternative or combination strategies.

A second generation of 7H-pyrrolopyrimidine analogues was made to explore how changing the size of the substituent influences how the PDK1 catalytic site works (O’Brien et al., 2014). These refined analogues showed that they were more effective than first-generation drugs. Compound 9j (4-methoxy) was the most promising lead, showing better kinase inhibition The research underscored the importance of the additional ring nitrogen in the purine-like scaffold, particularly for the most active compounds (9f and 9j), indicating the need for future structure–activity investigations (O'Brien et al., 2014).

### 4.2 Allosteric and dual-site modulation

Allosteric inhibitors target regions outside the ATP-binding site, including the PIF pocket, which is essential for substrate docking. These compounds deliver greater selectivity and minimize off-target effects compared to ATP-competitive inhibitors (Mingione et al., 2023).

#### 4.2.1 PIF-pocket inhibitors: RS2, PS210, PS48

PIF-pocket inhibitors constitute a uniquely mechanistic category of PDK1 modulators intended to interfere with substrate recognition instead of catalytic function (Rettenmaier et al., 2015). These compounds are designed based on the PDK1-interacting fragment (PIFtide) motif and function by occupying the hydrophobic PIF pocket, therefore inhibiting the docking of AGC kinase substrates such as S6K, RSK, and SGK (Sacerdoti et al., 2023). This technique provides an appealing pathway to improved selectivity, as the PIF pocket is structurally less conserved than the ATP-binding site and is specifically utilized by PDK1 to orchestrate downstream signaling outputs (Sacerdoti et al., 2023).

The initial proof-of-concept for this method was demonstrated by the diaryl sulfonamide RS2, initially discovered by Rettenmaier and associates, which efficiently obstructs substrate recruitment in the PIF pocket and inhibits downstream AGC kinase activation. RS2 demonstrates functional synergy with ATP-competitive PDK1 inhibitors, reinforcing the notion that simultaneous disruption of catalytic activity and substrate binding may result in more sustained pathway inhibition than either strategy independently (Rettenmaier et al., 2014). Subsequent investigations found dicarboxylate-based PIF-pocket binders, including PS210; nonetheless, inadequate membrane permeability significantly restricted their cellular and *in vivo* effectiveness. The ester prodrug PS48 was created to improve cellular absorption; nevertheless, its rapid hydrolysis and metabolic instability limited its translational potential, highlighting ongoing pharmacokinetic issues related to this chemotype (Busschots et al., 2012).

Among PIF-pocket ligands, sulfonamide derivatives, particularly RS2 analogues, have proven to be promising due to their advantageous physicochemical features, including pKa values conducive to effective cellular penetration. Studies on Structure–activity relationship (SAR) and subsequent structural analyses have shown that these compounds can selectively modulate PDK1 without significantly affecting the kinome, hence supporting the efficacy of allosteric targeting strategies (Rettenmaier et al., 2015; Xu et al., 2019). Recent studies have emphasized PIF-pocket inhibition as a basis for next-generation PDK1 drug design, especially when incorporated into dual-site inhibitors or degradation-based strategies intended to address pathway redundancy and adaptive resistance (Fu et al., 2025; Kailasam Natesan et al., 2023).

Studies on PIF-pocket inhibitors collectively demonstrate the extensive therapeutic potential of allosteric modulation in targeting PDK1. Despite early compounds encountering challenges with bioavailability and metabolic stability, they established essential design concepts that still guide contemporary endeavors in selective, multi-modal PDK1 inhibition (Arencibia et al., 2017).

#### 4.2.2 Dual-site inhibitors

Dual-site inhibition has arisen as a logical and increasingly persuasive approach for targeting PDK1, driven by the structural and functional constraints of single-site inhibitors (Xu et al., 2024). These drugs are engineered to concurrently interact with the highly conserved ATP-binding cleft and the PDK1-interacting fragment (PIF) pocket, thus integrating catalytic inhibition with the disruption of substrate docking. By targeting both sites, dual-site inhibitors not only reduce kinase activity but also obstruct compensatory recruitment and phosphorylation of downstream AGC kinases, a critical mechanism contributing to resistance against ATP-competitive inhibitors alone. This dual interaction provides a method to improve both efficacy and specificity while attaining more sustained route inhibition (Bobkova et al., 2010; Fu et al., 2025; Raimondi et al., 2012).

Recent structural and biophysical investigations have robustly corroborated this methodology. High-resolution crystal structures and molecular dynamics simulations demonstrate that the simultaneous interaction of the ATP site and PIF pocket stabilizes inactive kinase conformations and limits the conformational flexibility related to substrate accessibility and signaling adaptability (Wu et al., 2016). The PIF-pocket blocking significantly disrupts the docking of AGC kinases, including S6K, SGK, and RSK, hence diminishing downstream signaling, even in scenarios where partial ATP-site inhibition might typically be circumvented. This molecular understanding elucidates why dual-site methods are anticipated to surpass traditional ATP-competitive inhibitors in malignancies marked by pathway redundancy and adaptive feedback within the PI3K–AGC kinase network (Bobkova et al., 2010; Busschots et al., 2012; Fu et al., 2025; Raimondi et al., 2012; Xu et al., 2024).

Recent advancements in medicinal chemistry have highlighted the translational potential of dual-site PDK1 modulators. Recent evaluations emphasize novel chemotypes that combine ATP-site pharmacophores with PIF-pocket-binding motifs, utilizing structure-guided design to enhance binding shape, residence time, and selectivity profiles (Xu et al., 2024). These advanced inhibitors are especially appealing in carcinogenic contexts where PDK1 serves as both a catalytic kinase and a scaffolding hub, as they more efficiently limit both enzymatic activity and substrate reprogramming. Consequently, dual-site inhibition is a transformative approach to mechanism-aligned, multi-dimensional targeting of PDK1, establishing a conceptual basis for future therapeutic advancements, including the incorporation of degrader technologies and strategic combination methodologies (Fu et al., 2025; Mulpuri et al., 2025; Sestito et al., 2021).

### 4.3 Emerging multi-target approaches

To address pathway redundancy and drug resistance, multi-target strategies have been developed (Haynes and Manogaran, 2025). These strategies inhibit PDK1 along with complementary kinases, such as PI3K or Aurora-A. Pyridonyl compounds targeting the DFG-out conformation have shown efficacy against glioblastoma stem cells (Sestito et al., 2016). Heterocyclic inhibitors from companies like Sunesis and Biogen attack both ATP and PIF sites at the same time. These multi-target agents are efficacious against resistant tumors and frequently function independently of AKT signaling (Daniele et al., 2017; Park et al., 2023).

The multi-target approach reflects a growing understanding that single-target inhibition is often inadequate. The adaptability of cancer signaling networks undermines such approaches. Combining structural and mechanistic knowledge helps scientists come up with smart ways to make powerful, selective next-generation inhibitors. These strategies offer promise for overcoming resistance and improving clinical outcomes (Mulpuri et al., 2025; Xu et al., 2019).

The chemical diversity presented in Table 1 and Figure 4 provide a robust basis for the ongoing enhancement of PDK1-targeted therapeutics and demonstrates the progression of medicinal chemistry approaches in the domains. Initial ATP-competitive inhibitors like BX-795 and BX-912 exhibited significant biochemical potency but were constrained by extensive polypharmacology and inadequate translational applicability, while subsequent compounds such as GSK2334470 attained enhanced selectivity via structure-guided optimization, though they lacked sufficient *in vivo* efficacy (Jug and Ilaš, 2025). Concurrently, allosteric PIF-pocket inhibitors like RS1 and the dicarboxylate-derived PS210/PS48 family demonstrated the potential for selectively obstructing substrate docking while also exposing ongoing issues concerning permeability and metabolic stability (Busschots et al., 2012). Recent advancements in dual-site inhibitors, such as pyridonyl and hybrid chemotypes, combine ATP-site interaction with PIF-pocket inhibition to diminish both catalytic function and compensatory substrate acquisition (Jug and Ilaš, 2025). The scaffold classes collectively illustrate that a singular binding mode is inadequate for completely inhibiting PDK1 signaling in various biological contexts; instead, the comparative insights from Table 1 underscore the necessity for mechanism-informed, multi-modal design strategies that harmonize potency, selectivity, and drug-like characteristics to address pathway redundancy and enhance clinical translatability (Mansi et al., 2020).

**TABLE 1:** Classes of PDK1 inhibitors with representative compounds.

Class of inhibitor	Compound	IC_50_	Binding mode	Notes	References
ATP-competitive inhibitors	BX-795	6 nM	ATP-binding pocket (hinge, Ser94, Ala162)	Broad activity, also inhibits TBK1/IKKε	Tamgüney et al. (2008), Yu et al. (2020)
	BX-320	30 nM	ATP-binding pocket	Early tool compound, limited selectivity	Drewry et al. (2022), Feldman et al. (2005)
	BX-912	26 nM	ATP-binding pocket	Potent, but polypharmacology issues	Bai et al. (2020), Feldman et al. (2005)
	GSK2334470	10 nM	ATP-binding pocket (highly selective)	Gold-standard selective inhibitor	Medina (2013), Najafov et al. (2011), Schulze et al. (2016)
Allosteric PIF pocket inhibitors	RS1 (diaryl sulfonamide)	∼200 nM	PIF pocket (protein–protein interface mimic)	Cell-penetrant, synergizes with ATP-site inhibitors	Rettenmaier et al. (2014)
	PS210 (diaryldicarboxylate)	∼300 nM	PIF pocket	Poor permeability, improved with ester prodrug PS423	Busschots et al. (2012)
Indolinone-based inhibitor	BX-517	2–20 nM	ATP-binding site (hinge, Lys111, Thr222)	Urea substitution improves potency	Garcia-Echeverria and Sellers (2008), Islam et al. (2007b), Tokala et al. (2022)
Allosteric PH-domain antagonist/non-ATP-site modulator of PDK1	2-O-Bn-InsP5	26 nM	Interacts with the PDK1 PH domain, inhibiting it and PLCγ1’s recruitment to the membrane.	This binding selectively obstructs the activation of PDK1 substrates by altering their spatial location.	Raimondi et al. (2016)
Dual-site inhibitors	Sunesis pharmaceuticals, Inc. pyridinonyls	20–100 nM	ATP pocket + PIF pocket	Dual mechanism, effective in resistant cancers	Barile et al. (2012), Peifer and Alessi (2008)
DFG-out inhibitors Dual inhibition PDK1 and Aurora	SA16 (pyridonyl derivative)	Low µM	DFG-out conformation	Activity in glioblastoma stem cells	Daniele et al. (2017), Sestito et al. (2018), Sestito et al. (2021)
Quinazolin-12-ones	Compound 3b	−10.4 kcal/mol docking score	ATP pocket (Ala162 interactions)	Strong binding, BBB penetration, favorable ADMET	Irazoqui et al. (2023), Kailasam Natesan et al. (2023)

The structural and mechanistic diversity of PDK1 inhibitors demonstrates the advancement of multi-target design strategies (Arter et al., 2022). Figure 4 Representative inhibitors bind to various regulatory pockets, including the ATP-binding site, PIF pocket, PH domain, and DFG-out conformation, showing the viability of orthosteric, allosteric, and dual-site targeting. ATP-competitive ligands like GSK2334470 set standards for potency (Castel et al., 2016). Molecules such as 2-O-Bn-InsP_5_ and RS1 demonstrate that non-ATP binding domains can achieve selectivity and modulated protein interactions (Barile et al., 2012). DFG-out inhibitors and hybrid scaffolds exemplify new “dual-geometry” strategies that stabilize inactive conformations by interacting with accessory domains (Xu et al., 2024). These approaches could overcome pathway redundancy and drug resistance. Together, these structures highlight how rational polypharmacology through multi-target engagement or hybrid binding broadens the therapeutic landscape for PDK1 inhibition beyond traditional single-site models.

## 5 Medicinal chemistry and ADMET considerations

Developers of PDK1 inhibitors face significant challenges because ATP-binding sites remain highly conserved among kinases (Arter et al., 2022). Candidate drugs must robustly engage targets, maintain metabolic stability, efficiently enter cells, and exhibit favorable safety profiles (Badea et al., 2022).

### 5.1 SAR and scaffold optimization

Structure–activity relationship (SAR) studies of BX-series compounds (BX-320, BX-795, BX-912, BX-517) illustrate that core-molecule design is key to overcoming these issues (Abeyrathna and Su, 2015). Specifically, all inhibitors in this series share an N-phenylpyrimidin-2-amine core and, with 5-urea groups, form critical hydrogen bonds with Lys111 and Thr222. Notably, BX-517 is more potent due to stronger interactions with these residues (Feldman et al., 2005).

Subsequent systematic modifications of BX-517, including pyrrole C-4′ substitutions, generated derivatives (7b, 7d, 16, 17) with enhanced solubility and improved pharmacokinetics. These changes also led to an extended half-life. Nevertheless, some compounds continued to display limited microsomal stability. This incident illustrates the trade-offs involved in optimizing multiple pharmacological properties at once (Islam et al., 2007b).

Beyond the BX-series, other compound classes use distinct structural features for better selectivity and stability. For example, Sunesis’ pyridinyl compounds carry a difluorobenzyl carboxamide group, which enhances interactions with Ala162 and Ser160. Similarly, quinazolin-12-one compounds, such as 3f, cross the blood–brain barrier, bind less to plasma protein, and show low toxicity. Together, these features demonstrate how rational scaffold design can optimize drug properties (Hossen et al., 2015; Sestito et al., 2016).

### 5.2 Selectivity and pharmacokinetic challenges

Achieving selectivity for PDK1 inhibitors remains a major challenge. Molecules must distinguish among similar kinase active sites while minimizing off-target effects. BX-series analogs are effective but are linked to higher toxicity *in vitro*. In contrast, quinazolinones and sulfonamides such as RS1 offer better selectivity and oral bioavailability (Rettenmaier et al., 2014; Xu et al., 2019).

In addition to selectivity, optimizing pharmacokinetics requires detailed ADMET profiling. For example, while carboxylate compounds like PS210 need ester prodrugs to improve membrane permeability, these prodrugs are often rapidly hydrolyzed, thereby reducing plasma half-life (Wilhelm et al., 2012). In contrast, sulfonamides have favorable ionization properties, which aid absorption and systemic exposure. However, dicarboxylates remain problematic due to poor stability and short plasma residence times (Sadeghian et al., 2025; Wang et al., 2024). Thus, medicinal chemistry must optimize potency, metabolic stability, solubility, permeability, and *in vivo* durability to create clinically relevant candidates.

### 5.3 Drug-likeness and safety profiles

In the development of PDK1 inhibitors, both efficacy and safety are very important. Lipinski, Veber, Ghose, and Brenk’s classical guidelines examine molecular size, hydrogen bonding, lipophilicity, and structural alerts associated with safety risks. Modern design strategies, such as altering the core structure, targeting multiple sites, and using AI, are accelerating the search for better inhibitors. Inhibiting PDK1 at both the ATP-binding site and the PIF pocket simultaneously increases potency and reduces off-target toxicity. This dual blockade stabilizes inactive conformations and disrupts substrate docking, making the pathway more selective and safer for drug targeting (Chen et al., 2017; Irazoqui et al., 2023; Peifer and Alessi, 2008). Table 2 presents the main medicinal chemistry parameters and (SAR) trends that have led to improvements in PDK1 inhibitors. It emphasizes the need to balance molecular weight, polarity, lipophilicity, and ionization to achieve optimal permeability, selectivity, and metabolic stability. These principles are crucial for converting potent scaffolds into clinically viable, safe drug candidates.

**TABLE 2:** Medicinal chemistry insights: SAR trends, permeability, ADMET considerations.

Property/Issue	Desirable range/Approach	Design tip
Molecular weight	<500 Da (≤600 for dual-site)	Rigidify; avoid flexible linkers
cLogP	1–4	Add polar handles if > 4
PSA	<90 Å^2^ for cell permeability	Reduce HBD/HBA, use intramolecular H-bonds
HBD	≤3	Convert donors to H-bond acceptors if the permeability low
Ionizable groups	Sulfonamides (pKa ∼6–7) preferred over carboxylates	Use ester prodrugs for carboxylates only if stable
CYP inhibition	Minimal (IC50 > 10 µM)	Remove planar polyaromatics; block metabolic hotspots
P-gp efflux	Low	Reduce polarity and bulky amphiphilicity
PPB	Moderate (fu > 0.01)	Reduce lipophilicity; add polar surface
Ames/genotox	Negative	Avoid nitroaromatics and structural alerts
Selectivity	>100-fold vs. related AGC kinases ideally	Target unique hinge/gatekeeper features and PIF pocket

## 6 Therapeutic implications

Targeting PDK1 is a promising strategy for treating multiple cancers and other diseases. PDK1 is a central regulator of AKT and other AGC kinases. It drives oncogenic signaling linked to cell proliferation, survival, therapy resistance, and stem cell maintenance.

### 6.1 Potential therapeutic targets related to PDK1

Multiple therapeutically relevant signaling networks converge on phosphoinositide-dependent kinase 1 (PDK1), positioning it as a pivotal target in cancer treatment. PDK1 serves as a vital signal integrator in the PI3K–AKT–mTOR pathway, operating downstream of phosphoinositide production. The aberrant activation of this axis—caused by PIK3CA mutations, PTEN loss, or hyperactive receptor tyrosine kinases—constitutes one of the most prevalent oncogenic occurrences across various malignancies, and PDK1 inhibition provides a method to diminish pathway output regardless of specific upstream alterations (Arsenic, 2014; Castel et al., 2016; Maurer et al., 2009). Recent findings indicate that PDK1 dependence signifies pathway flux rather than static genetic alterations, thereby endorsing its targeting in PI3K-addicted cancers that exhibit resistance to AKT-centric therapies (Peng et al., 2025).

In addition to traditional PI3K signaling, PDK1 interacts with the RAS–MAPK pathway, especially in tumors with RAS and BRAF mutations. Genetic or pharmacological inhibition of PDK1 hinders tumor development, invasion, and metastasis in melanoma and pancreatic cancer models, highlighting its essential involvement even in MAPK-dominant environments (Ferro, 2014). This functional crosstalk is supported by findings that PDK1 maintains ERK-driven oncogenic activity via AKT-independent AGC kinases, such as RSK and SGK, thus facilitating signaling plasticity and adaptive resistance (Castel et al., 2016).

PDK1 regulates oncogenic transcriptional programs, particularly through the PDK1–PLK1–MYC axis, which stabilizes MYC and maintains proliferative and metabolic characteristics linked to aggressive illness and therapeutic resistance (Tan et al., 2013). Furthermore, PDK1 signaling influences not only tumor-intrinsic effects but also modulates the tumor microenvironment by regulating endothelial function, immune cell activation, and inflammatory signaling, with emerging evidence indicating its roles in immune metabolism and response to immunotherapy (Balkwill et al., 2012; Quail and Joyce, 2013). These actions collectively establish PDK1 as a systems-level regulator, whose targeting may provide distinct therapeutic advantages across genetically and microenvironmentally varied malignancies, as can be seen in Figure 3 (Gagliardi et al., 2018).

### 6.2 Preclinical and translational evidence

Preclinical studies provide compelling evidence for the therapeutic potential of PDK1 inhibition in cancer. In PTEN-deficient tumors, PDK1 inhibition prevents AKT activation and hinders anchorage-independent growth (Lu et al., 2010). These are characteristics typical of aggressive cancer phenotypes (Keenan Natesan et al., 2023). In breast cancer, PKD1 overexpression is linked to resistance to trastuzumab, tamoxifen, and PI3K inhibitors. Pharmacological inhibition of PDK1 restores sensitivity to these treatments and decreases tumor cell viability (Emmanouilidi and Falasca, 2017).

Furthermore, PDK1’s function encompasses neuroblastoma, where its inhibition, alongside AKT inhibitors like MK-2206, amplifies cytotoxicity, highlighting PDK1’s involvement in survival signaling pathways. In glioblastoma, the concurrent inhibition of PDK1 and Aurora-A yields superior antiproliferative effects compared to monotherapy This study illustrates the prospective advantages of concurrently targeting many signaling pathways to surmount compensatory mechanisms and improve therapeutic efficacy (Sestito and Rapposelli, 2019).

The development of combination and biomarker-driven therapies represents a pivotal strategy for PDK1-targeted interventions. PDK1 inhibitors have been found to synergize with various agents, including ATP-competitive AKT inhibitors, mTOR inhibitors, and PI3K inhibitors, thus suppressing oncogenic signaling and mitigating the activation of compensatory pathways. Recent research efforts have focused on dual targeting the PDK1 and SGK1 pathways to address challenges posed by PI3K-resistant tumors, as outlined in patent literature (Najafov et al., 2011).

Biomarkers such as phosphorylated Ser241 (p-Ser241), which indicate PDK1 activation, may help identify patient populations that would benefit from PDK1-targeted therapies. Furthermore, inhibiting PDK1 has been shown to sensitize cancer stem cells to standard treatments, potentially reducing cancer recurrence and metastasis (Nagashima et al., 2011; Park et al., 2023). These findings underscore the need to integrate PDK1 inhibitors into biomarker-driven, rational combination regimens to maximize therapeutic benefits.

In conclusion, summarized data across diverse cancer models (Table 3) indicate that both pharmacological and genetic inhibition of PDK1 attenuates malignant phenotypes. Specifically, in PTEN-deficient breast cancer models, PDK1 inhibitors (e.g., BX-320, GSK2334470) have been shown to reduce anchorage-independent growth and induce apoptosis while concurrently decreasing p-AKT (Thr308) and p-S6K signaling pathways. In neuroblastoma, PDK1 inhibition not only reduces cell viability but also resensitizes cells to AKT inhibitors, underscoring its role in overcoming therapeutic resistance. Furthermore, in glioblastoma stem-like cells, DFG-out PDK1 inhibitors (e.g., SA16) diminish neurosphere formation and invasion, particularly in combination regimens, highlighting the promising translational potential of targeting PDK1 in aggressive and resistant tumor contexts.

**TABLE 3:** Preclinical outcomes of PDK1 inhibition in different cancers.

Cancer type	Model (typical)	Representative compound(s)	Primary preclinical outcomes	Notes/Mechanistic readouts	References
Breast (PTEN-deficient)	MDA-MB-468, soft-agar, xenograft	BX-320, BX-series, GSK2334470	↓colony formation in soft agar; ↑apoptosis; tumor growth delay (genetic models)	Strong sensitivity in anchorage-independent assays; ↓ p-AKT (Thr308), p-S6K	Emmanouilidi and Falasca (2017), Wang et al. (2022), Yang et al. (2017)
Prostate (PTEN-null)	PC-3, spheroids, xenograft	GSK2636771	↓anchorage-independent growth; induced apoptosis	Selective effects in transformed cells vs. normal	Turnham et al. (2020)
Neuroblastoma	MK-2206-resistant and parental lines	BX-795 GSK2334470	Resensitization to AKT inhibitor; ↓ viability	Suggests role in AKT-inhibitor resistance	Chilamakuri et al. (2022), Qi et al. (2015)
Glioblastoma	Stem-like cell cultures, xenograft	SA16	↓neurosphere formation; reduced invasion; improved xenograft response when combined	DFG-out binders active vs. stem-like cells; CNS penetration required for brain tumors	Daniele et al. (2017), Sestito et al. (2016), Sestito et al. (2018)
Hematologic malignancies	BCR-ABL models, cell lines	PDK1 – inhibitors	↓survival in select lines; apoptosis induction	Some activity independent of AKT status	Nagashima et al. (2011), Quintás-Cardama and Cortes (2009)
Multiple tumors (lung, breast, ovarian, gastric, and pancreas)	Cell lines, xenografts	Multiple (tool compounds)	↓migration/invasion; tumor growth delay (combo)	Quinazolinones show promising PK/BBB *in silico*/*in vitro*	Noser et al. (2020)
PIF-pocket allosteric studies	Cell lines (various)	Quinazolinones (3f), pyridinonyls RS1 (diaryl sulfonamide), PS210/PS423	Block substrate docking; ↓S6K activation; synergy with ATP-site inhibitors	RS1: cell-permeant; PS210 needs prodrug PS423	Busschots et al. (2012)

### 6.3 Potential beyond oncology

PDK1 inhibition is currently being investigated beyond oncology, expanding its therapeutic applications. Research indicates that siRNA-mediated silencing of PDK1 can effectively diminish responses in ocular inflammation and allergy models, pointing towards PDK1’s role in immune regulation (Feldman et al., 2005). The structural distinctions observed between human and fungal PDK1 offer potential for creating selective antifungal drugs that minimize toxicity to host cells. Additionally, CNS-penetrant PDK1 inhibitors, particularly quinazolinone derivatives, present therapeutic potential for brain tumors and possibly neurodegenerative diseases by modulating PDK1-dependent survival pathways (Sestito and Rapposelli, 2019). These developments underscore the PDK1 utility as a pharmacological target across a range of diseases.

Furthermore, Table 4 furnishes a comprehensive overview of various PDK1 inhibitors explored in both preclinical and clinical realms, detailing their chemical classifications, mechanisms of action, and stages of development. It reveals that the majority of these compounds are still in preclinical optimization, with only a limited number progressing to early translational or combination studies, highlighting ongoing challenges in achieving selectivity and favorable pharmacokinetic properties. The table encapsulates the progression from early ATP-competitive inhibitors to contemporary dual-site and allosteric modulators, thereby illustrating the balance between the therapeutic potential of targeting PDK1 and the translational hurdles that remain. This analysis serves as a succinct connection between discovery research and clinical application.

**TABLE 4:** Some of preclinical PDK1 inhibitors.

Compound	Chemical class/Origin	Mechanism of action (PDK1)	Clinical/Preclinical status	Key outcomes	References
2-O-Bn-InsP5	Inositol-phosphate derivative	Binds PDK1 PH-domain; blocks PH-mediated PDK1 interactions	Preclinical/tool compound	Selective PH-domain inhibition; useful probe of PH-dependent PDK1 biology	Raimondi et al. (2016)
OSU-03012 (AR-12)	Celecoxib-derived small molecule	PDK1 inhibitor; induces ER/mitochondrial stress, interferes with PI3K/PDK1/AKT	Phase I clinical trial (AR-12) completed	Well tolerated in Phase I; preclinical antitumor activity across models	Mateo et al. (2013)
GSK2334470 (GSK-470)	Aminopyrimidine/aminoindazole (GSK)	Highly selective ATP-competitive PDK1 inhibitor; blocks T-loop phosphorylation of AGC kinases	Preclinical/chemical probe	Sub-10 nM biochemical potency; cytotoxic in multiple myeloma; substrate-selective effects on AKT under high PIP_3_	Emmanouilidi et al. (2019), Medina et al. (2011), Najafov et al. (2011)
SNS-229/SNS-510	Proprietary heterocyclic inhibitors (Sunesis/SNS series)	Inactive-state/DFG-like binders targeting PDK1 conformations	Preclinical/proprietary	Induce apoptosis and tumor regression in some models; reported to bind inactive conformer	Hansen et al. (2015)
UCN-01 (7-hydroxystaurosporine)	Staurosporine derivative	Broad-spectrum ATP-site kinase inhibitor including PDK1	Terminated	Clinical activity with combinations; nonselective with multiple off-targets	Komander et al. (2003)
BX-795/BX-912/BX-320	Imidazopyrimidine/aminopyrimidine (BX series)	ATP-competitive PDK1 inhibitors; block T-loop phosphorylation	Preclinical	Nanomolar enzymatic potency; strong activity in PTEN-null/Akt-hyperactive models; some polypharmacology (BX-795 inhibits TBK1)	Balendran et al. (2000), Najafov et al. (2011)
PHT-427	PH-domain binder (small molecule)	Binds PH domains of Akt and PDK1; disrupts membrane recruitment	Preclinical/translational	Dual PH-domain inhibition; *in vivo* antitumor activity reported	Meuillet et al. (2010)
PDK1-IN-RS2 (RS2)	Diaryl small molecule (PIF-pocket allosteric binder)	Binds the PIF-pocket (substrate docking site); substrate-selective inhibition	Preclinical/tool compound	Disrupts docking of PIF-dependent substrates; synergizes with ATP-site inhibitors	Busschots et al. (2012), Wilhelm et al. (2012)
PS48/(R)-PS210	Allosteric PIF-pocket modulators/activators	PIF-pocket binders (some series are activators or prodrugs)	Preclinical/tool compounds	Used to map allosteric modulation; some prodrug strategies (PS423) improved permeability	Bobkova et al. (2010), Busschots et al. (2012), Wilhelm et al. (2012)

## 7 Current challenges and future perspectives

Despite extensive research, no selective PDK1 inhibitor has entered clinical trials. Ongoing biological, chemical, and translational challenges hinder clinical translation, revealing the complexity in targeting this kinase.

### 7.1 Obstacles to clinical translation

The clinical translation of PDK1-targeted therapies has been impeded by a confluence of medicinal chemistry, biological, and translational challenges. The principal challenges include insufficient selectivity and suboptimal pharmacokinetic properties of initial inhibitors, prevalent pathway redundancy and adaptive resistance within the PI3K–AGC kinase network, and the absence of validated prognostic biomarkers for efficient patient stratification (Baldi et al., 2025; Fu et al., 2025). The on-target toxicity associated with the critical physiological functions of PDK1 has limited treatment windows, highlighting the necessity for mechanism-aligned, biomarker-driven, and multi-modal approaches to realize the clinical potential of PDK1 inhibition (Domrachev et al., 2021).

#### 7.1.1 Restricted selectivity and binding site limitations

A primary challenge in the advancement of PDK1 inhibitors is the restricted selectivity due to the largely conserved ATP-binding site, which complicates the creation of molecules with an acceptable safety profile. Initial ATP-competitive inhibitors, such as BX-795 and its BX-series analogues, demonstrated extensive polypharmacology within the kinome, complicating the assessment of preclinical efficacy and raising apprehensions about off-target toxicity and translational reliability (Hossen et al., 2015; Peifer and Alessi, 2008). While allosteric and PIF-pocket modulators enhance target selectivity, these compounds frequently result in partial or context-dependent inhibition and necessitate meticulous optimization to attain sustained pathway suppression, especially amid adaptive signaling plasticity within the PI3K–AKT–mTOR network (Baldi et al., 2025; Medina, 2013; Mulpuri et al., 2025).

#### 7.1.2 Limitations of pharmacokinetic and drug-like properties

Pharmacokinetic limitations and inadequate drug-like characteristics further impede the clinical progression of PDK1 inhibitors. Numerous powerful drugs possess polar carboxylate or heteroaromatic structures that require prodrug methods to improve membrane permeability; nonetheless, rapid hydrolysis and metabolic instability often restrict systemic exposure and tumor infiltration (Carbone et al., 2023; Islam et al., 2007a; Islam et al., 2007b; Zhou et al., 2024). Highly selective tool compounds like GSK2334470 have strong biochemical target engagement but lack adequate *in vivo* anticancer efficacy, highlighting the ongoing disparity between enzymatic inhibition and therapeutic effectiveness (Carbone et al., 2023; Fu et al., 2025). These limitations collectively underscore the insufficiency of traditional small-molecule strategies when addressing a signaling hub characterized by intricate spatial control and non-catalytic scaffold roles.

#### 7.1.3 Redundancy in pathways and mechanisms of adaptive resistance

In addition to the constraints of medicinal chemistry, route redundancy and adaptive resistance mechanisms provide essential biological obstacles to the efficient targeting of PDK1. Pharmacological inhibition of PDK1 often results in cytostatic rather than cytotoxic effects due to compensatory activation of parallel AGC kinases, AKT-independent mTORC1 signaling, feedback loops from receptor tyrosine kinases, or the activation of alternative survival pathways such as YAP1 (Castel et al., 2016). These escape mechanisms are intensified by ATP-competitive inhibitors, which inadequately reduce PDK1’s non-catalytic scaffold functions and do not obstruct PIF-pocket-mediated substrate docking, thereby allowing residual signaling despite evident target engagement (Baldi et al., 2025; Fu et al., 2025).

#### 7.1.4 Absence of predictive biomarkers and patient stratification

The lack of proven predictive biomarkers and insufficient patient classification tools is equally restrictive. In contrast to actionable oncogenic drivers like EGFR, BRAF, or PIK3CA, activating mutations in PDPK1 are few (<1%), and neither total PDK1 expression nor isolated phosphorylation at Ser241 has been clinically verified as a companion diagnosis (Domrachev et al., 2021; Zhang et al., 2017). Extensive genomic and proteogenomic studies reveal that PDK1 reliance is significantly context-dependent, arising in cancers marked by PTEN loss, PIK3CA mutation, PDPK1 amplification, or lipid-induced hyperactivation. Nonetheless, these characteristics have not been methodically integrated into clinical trial design, resulting in the inclusion of physiologically unselected patient cohorts and a dilution of the treatment signal (Fu et al., 2025).

#### 7.1.5 On-target toxicity and limited therapeutic range

Ultimately, the on-target toxicity resulting from the pervasive physiological functions of PDK1 has created a restricted treatment window that has hindered clinical advancement. PDK1 is crucial for glucose metabolism, immune cell activation, and neuronal survival, and its systemic inhibition replicates the toxicities associated with PI3K inhibitors, such as hyperglycemia and immunological dysregulation (Baldi et al., 2025). As of now, no PDK1 inhibitor has progressed past early-phase clinical trials, highlighting the difficulty of effectively suppressing the pathway in malignancies without causing intolerable disturbance to normal tissue homeostasis (Domrachev et al., 2021).

### 7.2 Innovative design and polypharmacology

Future strategies emphasize rational design that unites selectivity, potency, and redundancy management. Polypharmacology targeting PDK1 with PI3K, mTOR, or Aurora-A offers a promising solution for compensatory signaling and can improve outcomes.

Structural insights enable targeting unique residues, such as Ala162 in the hinge. Stabilizing autoinhibited conformations using a linker mimetic provides an additional approach for modulating kinases. Biomarker-guided patient selection may enhance clinical efficacy, especially in tumors with elevated Ser241 phosphorylation. These developments could enable personalized therapy. AI-driven, structure-based design can accelerate the discovery of compounds with improved pharmacokinetics, selectivity, and potency (Mulpuri et al., 2025; Peifer and Alessi, 2008).

### 7.3 Applying AI to generating kinase inhibitors

Recent breakthroughs in computational chemistry and artificial intelligence have significantly enhanced kinase drug discovery by enabling the efficient investigation of vast chemical space and structure-based optimization (Yasuda et al., 2025). Structure-based drug design (SBDD), including molecular docking and molecular dynamics simulations, is extensively utilized to investigate kinase binding pockets, evaluate ligand-target interactions, and enhance lead compounds prior to their synthesis and biological testing. This method saves time and money compared to traditional methods (Wei and McCammon, 2024).

Machine learning–based virtual screening methods, such as deep learning and generative scoring functions, can accelerate the identification of potential inhibitors by predicting binding affinity and chemical features associated with activity. When used with multiconformational protein ensembles, these methods can also improve hit rates against dynamic targets (Loeffler et al., 2024). Another promising approach to suggesting new scaffolds that fit specific kinase pockets is to use chemical language models (CLMs) and reinforcement-learning frameworks to generate new designs from scratch. This combines structural and chemical constraints (Yin et al., 2024).

For PDK1, structure-based pharmacophore modeling and protein–protein docking have recently been used to identify allosteric pharmacophoric features in PDK1’s PIF pocket and to support virtual screening of different libraries. Met dynamics and free-energy analysis have confirmed possible binders (Vennila and Elango, 2024). Integrated workflows that combine docking with molecular mechanics have also become more common. Born surface area (MM-GBSA) rescoring and dynamics can analyze the stability and specificity of potential PDK1 ligands *in silico* (Kirubakaran et al., 2012; Sadeghian et al., 2025). While there have not yet been any reports of fully AI-generated, experimentally validated PDK1 inhibitors, the combination of generative design, physics-based screening, and machine learning-enhanced optimization provides a strong framework for accelerating the search for selective, potent PDK1 inhibitors in future studies.

## 8 Expert opinion

Recent advances in PDK1 biology, structural analysis, and medicinal chemistry highlight its promising potential and the challenges it poses as a therapeutic target. The development of selective ATP-site inhibitors, PIF-pocket allosteric ligands, and dual-site scaffolds has expanded options for modulating PDK1 activity and supports rationally designed combination strategies in oncology and other diseases.

### 8.1 Translational opportunities and limitations

PDK1 inhibitors will likely impact clinical practice as part of biomarker-guided combination therapies. Patients with PTEN loss or hyperactive PI3K signaling may respond to AKT inhibitors. Brain-penetrant scaffolds might help in CNS malignancies. Yet, clinical progress is slow (Raju et al., 2023). This situation is due to selectivity challenges, incomplete biomarker validation, and poor *in vivo* efficacy of current compounds.

Closing the gap between preclinical promise and clinical utility requires validated pharmacodynamic markers, such as p-Ser241 or substrate phosphorylation signatures, alongside proof-of-concept studies showing synergy in combination regimens. Without this, clinical integration of PDK1-targeted strategies will remain challenging.

### 8.2 Barriers to clinical translation of PDK1 inhibitors

Despite a robust biological foundation, the clinical application of PDK1-targeted therapeutics has been hindered by a confluence of chemical, biological, and translational obstacles, necessitating a comprehensive evaluation of the factors that have impeded success to date. While PDK1 (PDPK1) is infrequently altered, numerous extensive cancer genomics studies highlight its clinical significance via gene amplification and pathway reliance rather than traditional oncogenic mutation. The TCGA dataset indicates that PDPK1 copy-number gain or amplification occurs in approximately 5%–15% of breast, ovarian, lung, and head and neck squamous cell carcinomas, which is associated with heightened PI3K pathway activity and a poor prognosis (TCGA Research Network, 2020; Lui et al., 2013; He et al., 2021; Qi et al., 2025). Conversely, activating point mutations in PDPK1 is infrequent, constraining mutation-based patient classification and partially elucidating the unsuccessful outcomes of previous therapeutic studies that depended on expression rather than functional reliance (Zhang et al., 2017). Recent research indicates that indicators of pathway flux may more accurately predict sensitivity to PDK1 inhibition than static genetic changes. These factors encompass PTEN loss, PI3KCA mutation, increased PIP_3_ levels, and reliance on AKT-independent PDK1 substrates such as SGK3 or RSK, especially in cancers exhibiting diminished AKT signaling or metabolic reprogramming (Prabhu et al., 2025; Scortegagna et al., 2014; Scortegagna et al., 2015; Zhou et al., 2024). Recent mechanistic investigations suggest that phosphoinositide-mediated PDK1 conformational activation and persistent Ser241 phosphorylation, rather than overall protein levels, may characterize functional dependence on PDK1 signaling (Huang et al., 2024; Levina et al., 2022; Sacerdoti et al., 2023). The data collectively suggest that PDK1 represents a context-dependent vulnerability, necessitating sensible patient selection based on integrated lipid signaling, PTEN status, and downstream substrate activation measures, rather than only on PDPK1 expression or mutation.

### 8.3 Strategic directions for future research

Subsequent research in PDK1-targeted therapy must emphasize the creation of next-generation allosteric and dual-site inhibitors to enhance selectivity, maintain prolonged pathway suppression, and expand therapeutic windows. A thorough pharmacological assessment in genetically and phenotypically pertinent patient-derived models will be crucial for forecasting clinical efficacy and reducing translational failure. The early incorporation of comprehensive biomarker research initiatives in drug discovery pipelines is crucial for reasonable patient classification and the reduction of on-target toxicity in early-phase clinical evaluations. Collectively, these initiatives facilitate a significant shift from non-selective, ATP-competitive suppression to precision-focused, mechanism-aligned, and multi-modal therapeutic approaches (Roskoski, 2025; Wang et al., 2021).

In the forthcoming decade, rational polypharmacology strategies are anticipated to become a primary emphasis, integrating PDK1 regulation with inhibitors of PI3K, AKT, or analogous signaling pathways to mitigate route redundancy and adaptive signaling plasticity. Structural and signaling investigations increasingly endorse dual-site targeting paradigms, wherein simultaneous interaction with the ATP-binding cleft and rupture of the PIF-pocket stabilize inactive kinase conformations and inhibit the docking of downstream AGC substrates. These “dual-geometry” inhibitors possess the capability to inhibit both catalytic activity and compensatory substrate reprogramming, a prevalent resistance mechanism linked to single-site inhibitors. Concurrently, tailored protein degradation techniques, such as PDK1-directed PROTACs, signify a revolutionary approach by facilitating the removal of both catalytic and non-enzymatic scaffold activities that uphold signaling robustness and are unattainable by traditional small-molecule inhibitors.

Effective clinical translation will ultimately rely on the integration of sophisticated treatment modalities with biomarker-driven patient selection frameworks that emphasize functional pathway reliance rather than solely PDPK1 expression. Genomic factors, including PTEN loss, PIK3CA mutation, or PDPK1 amplification, along with lipid-dependent activation states evidenced by persistent Ser241 phosphorylation and synchronized activation of downstream AGC kinases, may function as dynamic markers of PDK1 dependency and therapeutic susceptibility (Emmanouilidi et al., 2019). Pan-cancer proteogenomic and functional dependency analyses indicate that pathway activation signatures, rather than singular genomic alterations, more precisely reflect PDK1 dependence, with CRISPR and RNAi screens revealing tumor subsets addicted to PI3K or amplified in RTK where PDPK1 is critical for survival. Expanding the exploration of PDK1 beyond oncology to include inflammatory, metabolic, and neurodegenerative diseases where PI3K–PDK1–AGC signaling regulates immune activation, metabolic balance, and neuronal survival highlights the kinase’s extensive pharmacological significance and advocates for its reclassification as a context-specific, multi-disease therapeutic target rather than a universal growth kinase.

### 8.4 Broader impact on precision medicine

Next-generation targeted protein degradation techniques such as proteolysis-targeting chimeras (PROTACs), molecular glues, and novel degrader–antibody conjugates (DACs) present an effective approach to overcome persistent challenges associated with kinase inhibition, especially for signaling hubs like PDK1. By eradicating the target protein instead of merely transiently inhibiting catalytic activity, these strategies possess the capacity to surmount conformational plasticity, disrupt non-enzymatic scaffold functions, and mitigate compensatory signaling networks that often undermine small-molecule inhibitors. The integration of PDK1-binding scaffolds into degrader platforms may facilitate enhanced and prolonged pathway inhibition in tumors exhibiting hyperactive PI3K–AKT signaling, consistent with recent clinical progress in kinase degradation noted for BTK, BRAF, and CDK family members (Békés et al., 2022; Li and Crews, 2022; Mullard, 2025).

It is essential to differentiate between direct degradation of PDK1 and indirect regulation of pathways through the degradation of upstream regulators. The newly identified SMART1 chemical does not act as a PROTAC that directly destroys PDK1; instead, it serves as a highly selective degrader of Smurf1, an E3 ubiquitin ligase that regulates PDK1–AKT signaling via post-translational mechanisms. SMART1 was discovered via the examination of a structurally varied PROTAC library and consists of a ligand that binds to Smurf1, targeting the HECT domain, an optimized linker, and a cereblon-recruiting component, leading to effective, CRBN- and proteasome-mediated degradation of Smurf1 in KRAS-mutant colorectal cancer models (Zhang et al., 2017). This distinction is crucial to prevent confounding pathway-level regulation with direct target engagement in the developing degradation literature.

Mechanistically, the loss of Smurf1 impairs PDK1 signaling indirectly by inhibiting PDK1 neddylation at Lys163, a change essential for the recruitment of SETDB1 and the assembly of the cytoplasmic cCOMPASS complex, which enables AKT membrane localization and Thr308 phosphorylation (Peng et al., 2025). The loss of this regulatory axis diminishes PDK1 Ser241 phosphorylation and weakens downstream PI3K–AKT pathway output without direct interaction with PDK1, indicating an extra layer of upstream regulation over PDK1 functional activation. SMART1 exhibits nanomolar antiproliferative activity in KRAS-mutant colorectal and pancreatic cancer cell lines, significant antitumor efficacy in both cell-derived and patient-derived xenograft models, and favorable tolerability, while demonstrating synergy with ATP-competitive PDK1 inhibitors. These findings collectively affirm the viability of targeting the PDK1 pathway indirectly through E3 ligase degradation as a therapeutic strategy, while highlighting that genuine PDK1-directed degraders are still in the nascent stages of preclinical development, with only recent studies initiating exploration of this area (Peng et al., 2025).

## 9 Conclusion

As a principal regulator of AGC kinases, PDK1 occupies a central role in oncogenic signaling. Early inhibitors demonstrated druggability; however, challenges with selectivity, pharmacokinetics, and pathway redundancy have hindered clinical translation. Advances in structural biology and medicinal chemistry, particularly allosteric modulation and dual-target approaches, are creating new opportunities. Incorporating PDK1 inhibitors into rational combination therapies guided by predictive biomarkers may realize their full therapeutic potential. Emerging applications for PDK1-targeted agents include allergies, antifungal infections, CNS diseases, and cancer. The next-generation of inhibitors may ultimately fulfill the clinical promise of PDK1 regulation by building on current knowledge.

Future strategies that combine PDK1-targeting chemotypes with novel modalities, such as PROTACs, DACs, and molecular glues, may address persistent challenges in selectivity and pharmacokinetics. These approaches promote catalytic degradation of PDK1, resulting in more robust and sustained pathway suppression (Békés et al., 2022; Xu et al., 2019). When combined with biomarker-driven patient selection, degraders and dual-site inhibitors could lead to a new generation of precision therapeutics that are better than traditional kinase inhibition.
